# Supplementary material for: Optimal Frequency for Seizure Induction With Electroconvulsive Therapy and Magnetic Seizure Therapy in Nonhuman Primates
Source: Biol Psychiatry Glob Open Sci. 2025 Feb 20;5(3):100471. doi: 10.1016/j.bpsgos.2025.100471 (PMC11985115; doi:10.1016/j.bpsgos.2025.100471)
Supplement: Supplemental Text and Figures S1–S13 [file mmc1.pdf]

SUPPLEMENTARY INFORMATION

Optimal Frequency for Seizure Induction With Electroconvulsive Therapy and Magnetic Seizure Therapy in Nonhuman Primates

Peterchev *et al.*

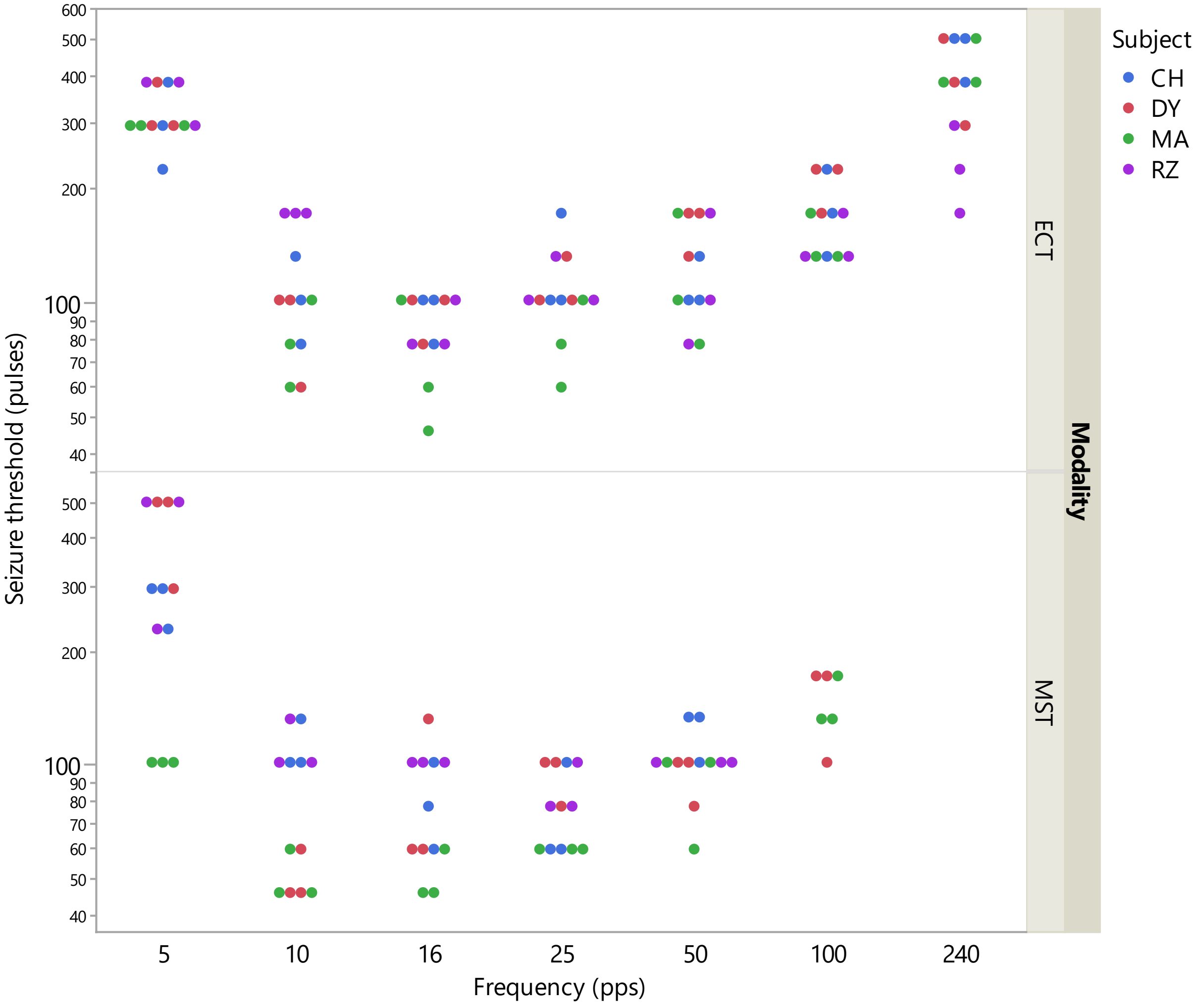

Figure S1. Individual seizure threshold (number of pulses) data corresponding to Figure 2A.

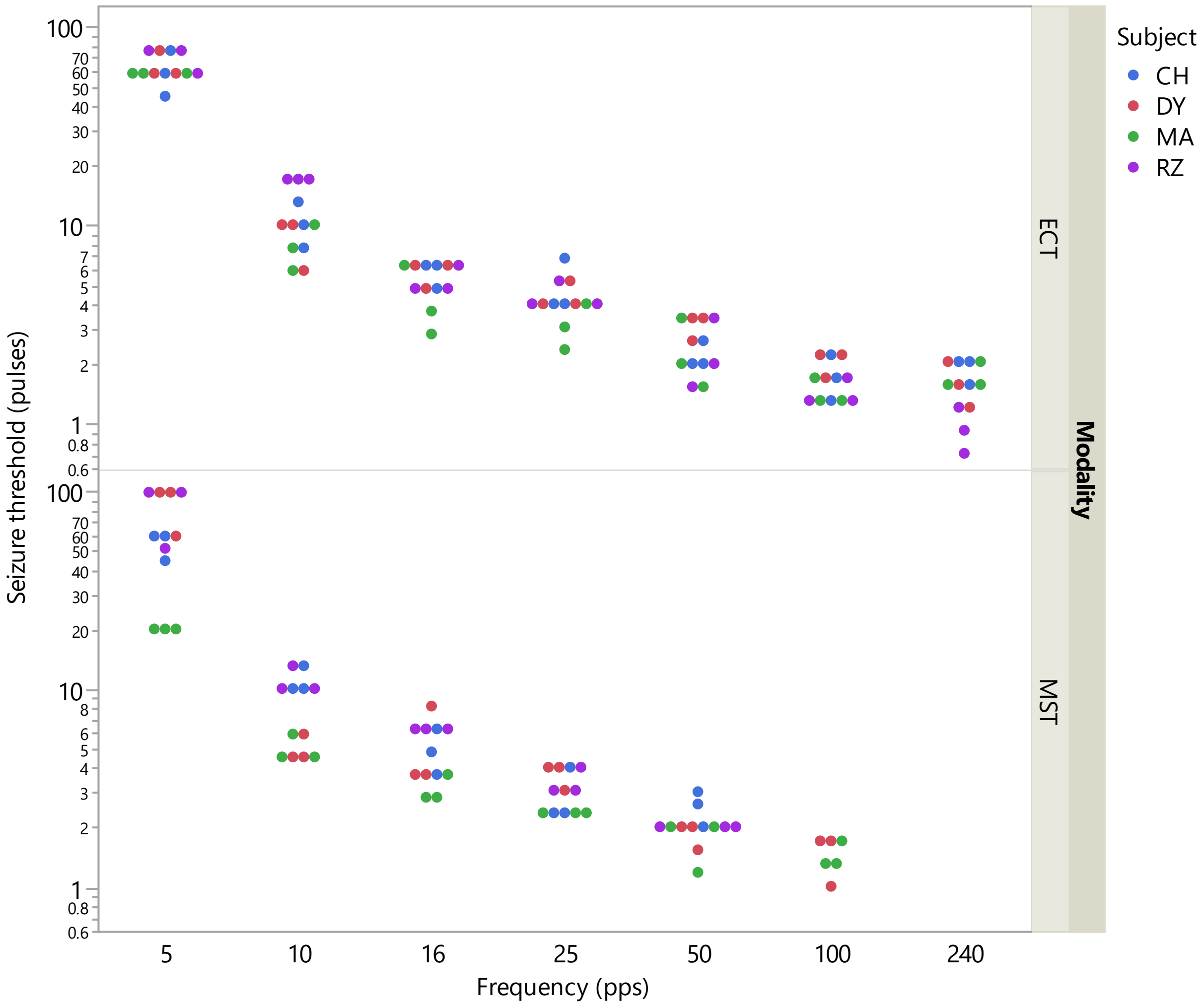

Figure S2. Individual seizure threshold (train duration) data corresponding to Figure 2B.

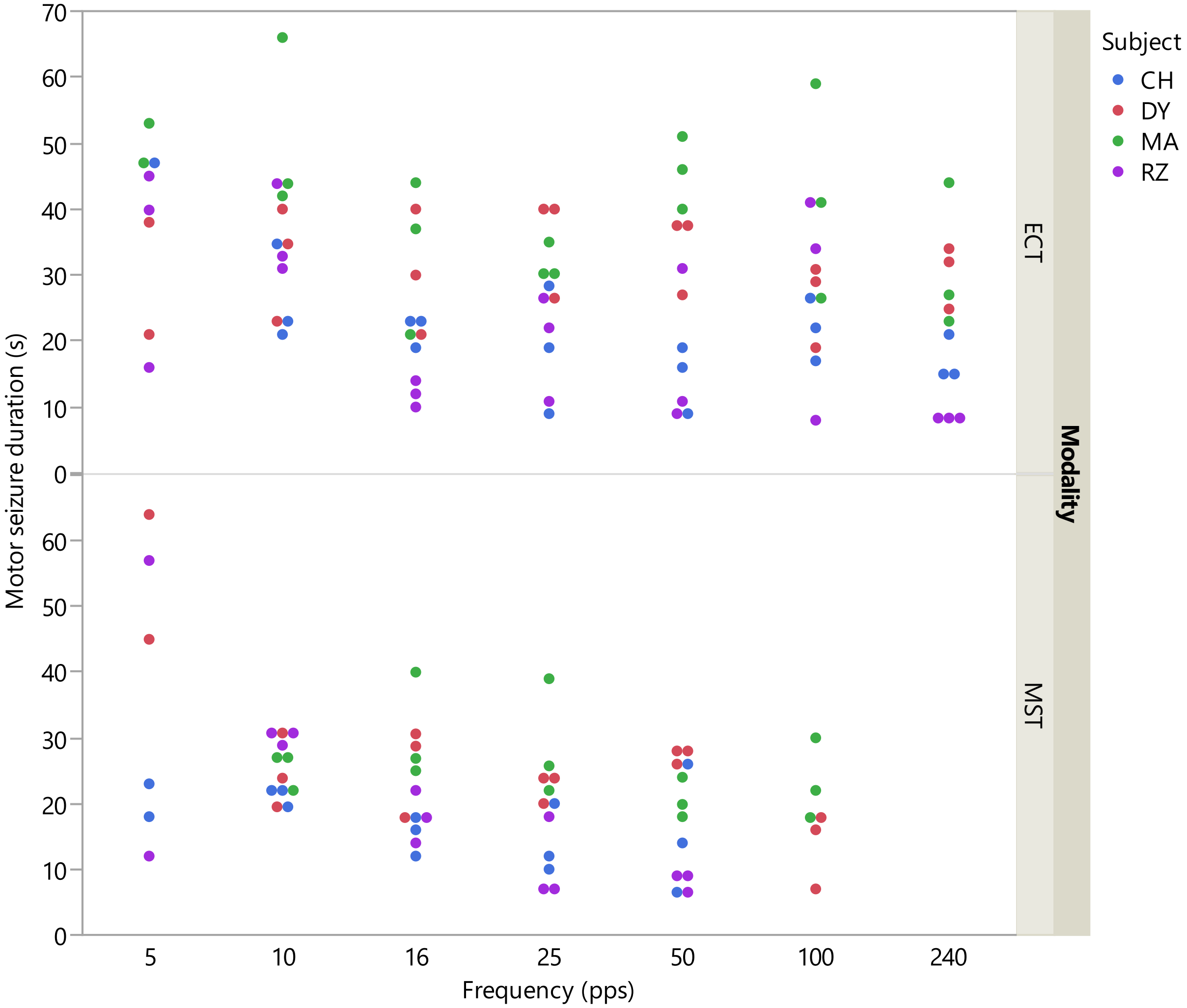

Figure S3. Individual motor seizure duration data corresponding to Figure 2B.

Qualitative seizure expression rating

Seizure rating methods

The visually observed strength of the motor seizure expression in the four limbs (separate for tonic and clonic phase) and face was rated on a qualitative scale of "none-weak-medium-strong." Seizure strength ratings were documented for 143 of the 150 sessions. These qualitative ratings are reported only descriptively, since they were not standardized and were conducted by various raters.

Seizure rating results

As expected, in the paralyzed limbs observed seizure strength was rated as "none" in the vast majority of sessions (94%–96%). In the face, 25.5%, 52.5%, and 22.0% of the seizures were rated as "none", "weak", and "medium", respectively. These ratings likely depend strongly on the depth of paralysis. In contrast, observed seizure strength in the unparalyzed arm was rated as "medium" for the vast majority (88%–89%) of seizures, as shown in Figures S4 and S5.

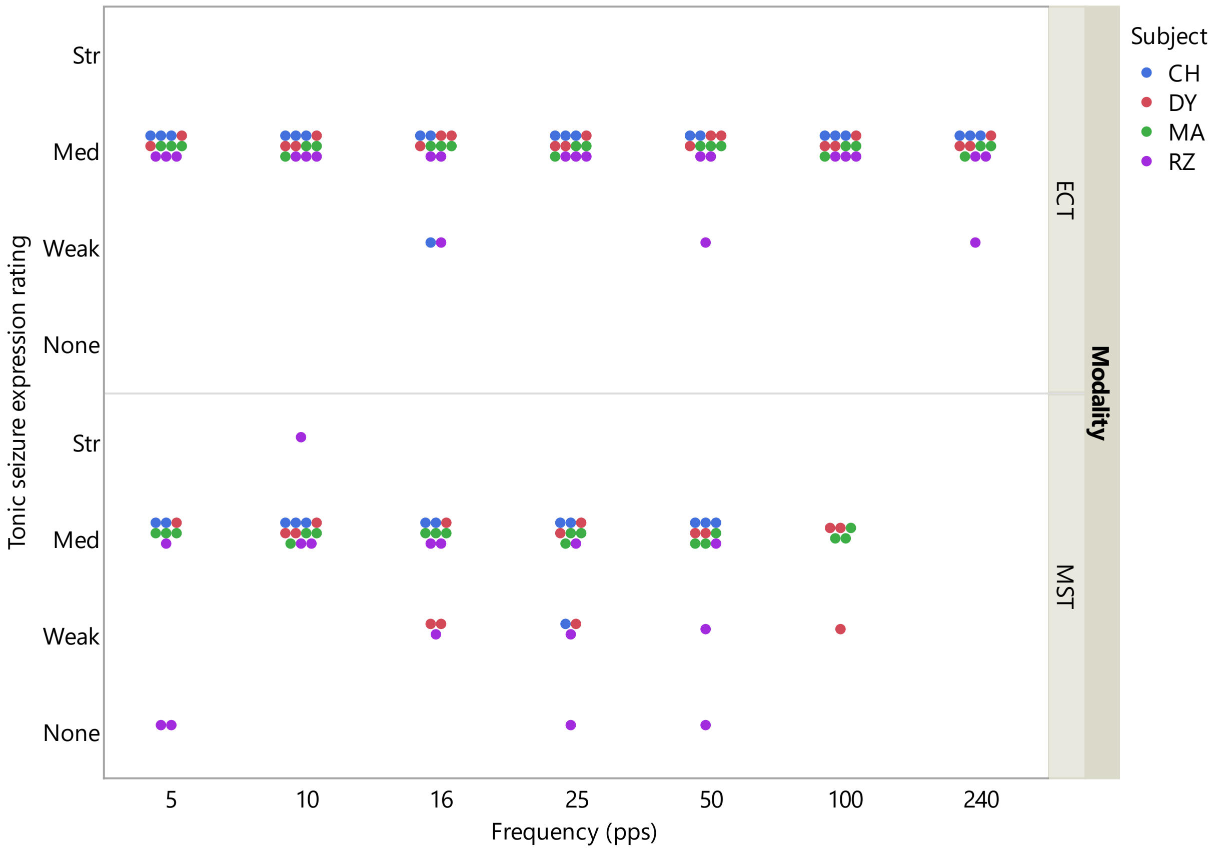

Figure S4. Tonic motor seizure expression rating for unparalyzed arm.

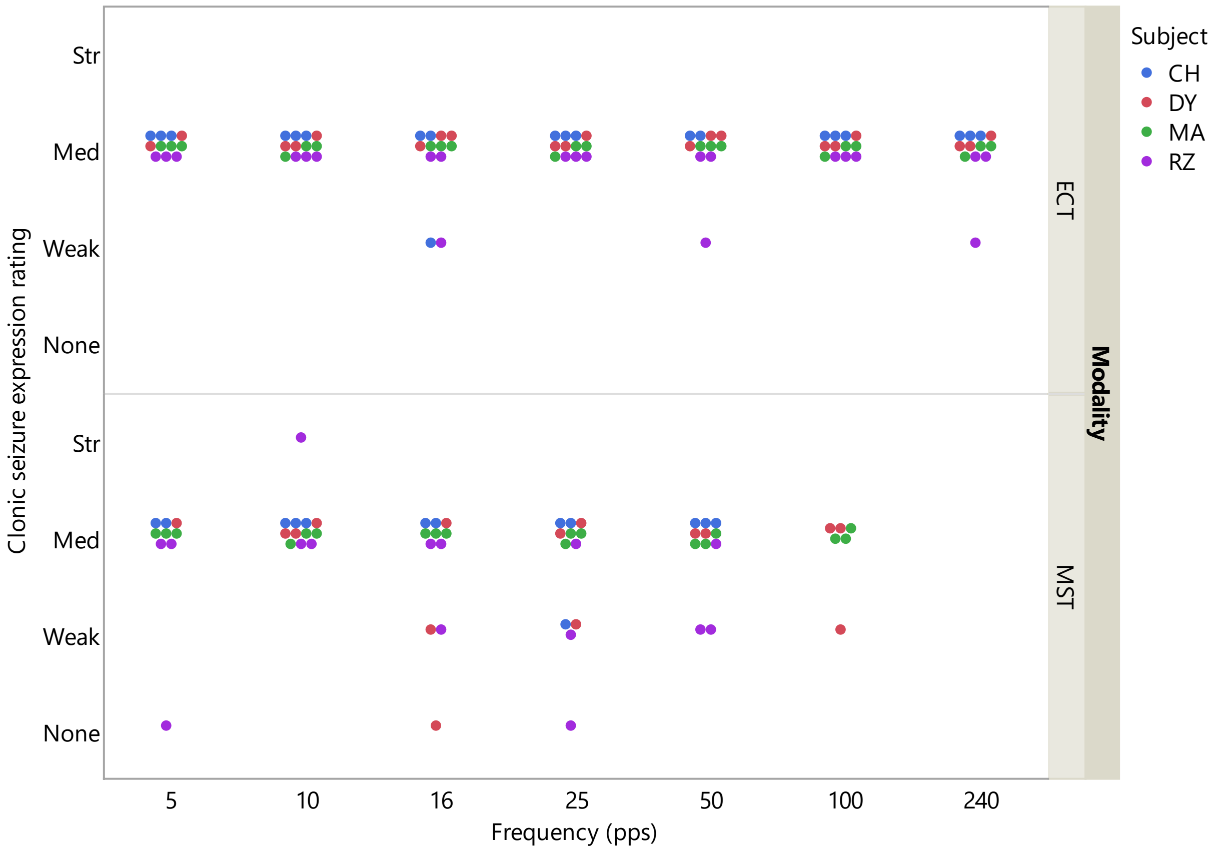

Figure S5. Clonic motor seizure expression rating for unparalyzed arm.

Electromyography (EMG)

EMG methods

Unlike EEG data, EMG signals can be collected and analyzed during the ECT and MST stimulus delivery, providing additional quantitative information about the seizure characteristics. EMG data collection was piloted during 17 of the ECT sessions from all seven stimulus frequency conditions (5 pps, n = 2; 10 pps, n = 2; 16 pps, n = 4; 25 pps, n = 2; 50 pps, n = 3; 100 pps, n = 2; 240 pps, n = 2). EMG signal was recorded with needle electrodes inserted in the first dorsal interosseous muscle, amplified with bioamp (BIOAMP-4, SA Instrumentation Co.), and digitized at 5 kHz sampling frequency (USB-6009, National Instruments). We extracted the EMG signal from 5 seconds before stimulus onset to 5 seconds after seizure termination. Tonic and clonic seizure duration was calculated based on methods of Conradsen et al. (1). Briefly, the EMG was resampled to 1024 Hz and decomposed into a high-frequency (HF) component (64–256 Hz) and a low-frequency (LF) component (2–8 Hz) using an 8-level decomposition with the Daubechies 20 (db20) wavelet. The HF/LF ratio was computed. The tonic-clonic transition was defined as the time where the HF/LF ratio drops to below 20% of its maximum (Figure S6).

EMG results

Seizure duration determined by EMG was consistent with the observation of motor activity (Figure S7A). There was a significant effect of stimulus frequency on tonic phase duration (log-transformed frequency, $R^2 = 0.297$, $p = 0.0238$), but the tonic phase proportion of the total seizure length did not change significantly with stimulus frequencies (Figure S8). This suggests that although frequency did affect the overall seizure duration, the split of the seizure in tonic and clonic components was not altered significantly.

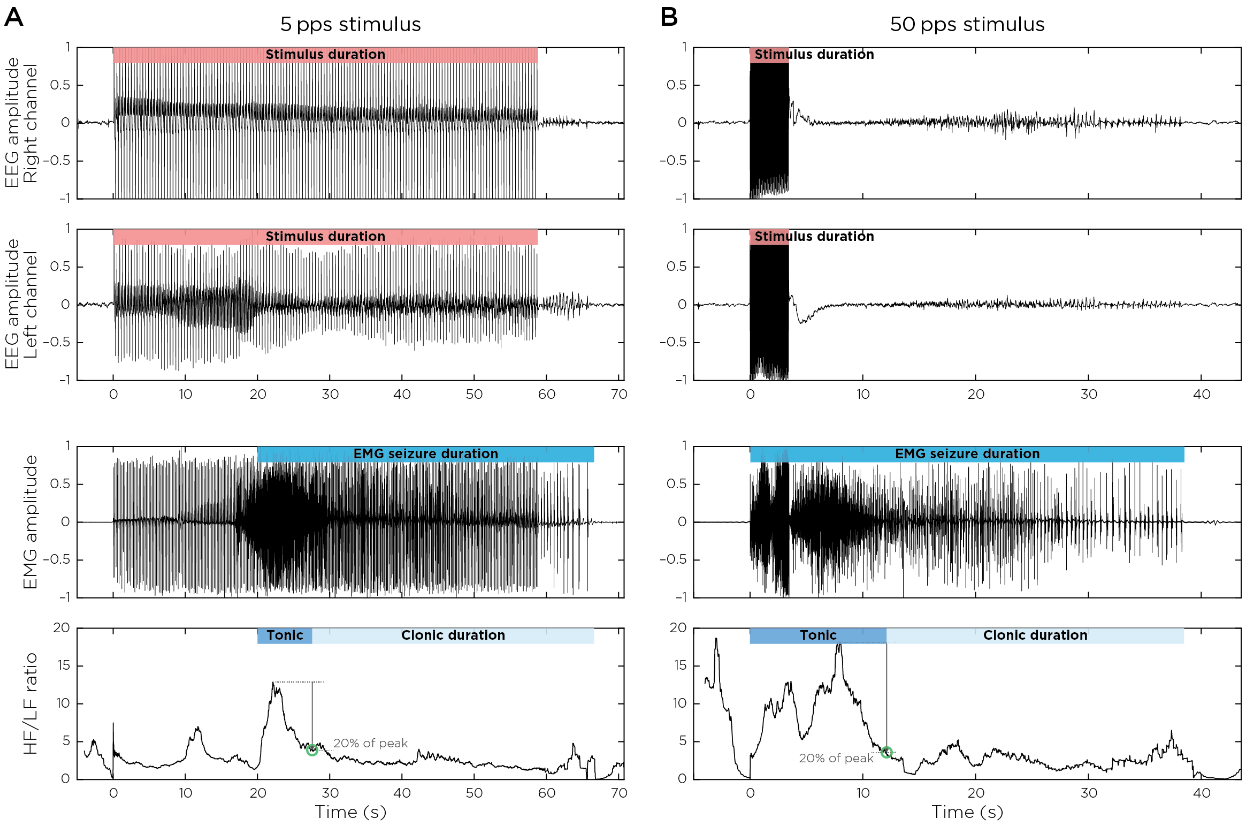

Figure S6. EMG recorded from one subject (MA) receiving ECT with (A) 5 pps and (B) 50 pps stimulus frequency. The top two rows show right and left normalized EEG channel amplitude. The third row shows normalized EMG signal from 5 seconds before stimulus onset to 5 seconds after seizure termination. The bottom panels show the corresponding high-frequency-to-low-frequency (HF/LF) EMG ratios. In the bottom panel, the blue bar differentiates the tonic and clonic seizure phases.

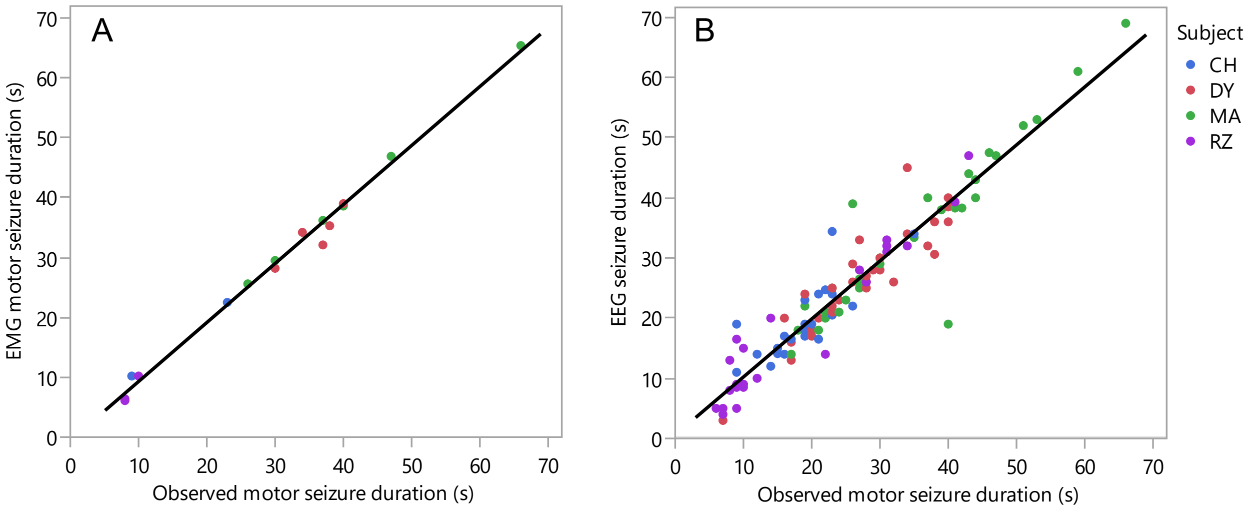

Figure S7. Correlation between observed motor seizure duration and (A) EMG motor seizure duration (R^2 = 0.992, p < 0.0001) and (B) EEG seizure duration (R^2 = 0.899, p < 0.0001). Colors denote subject and regression line is in black.

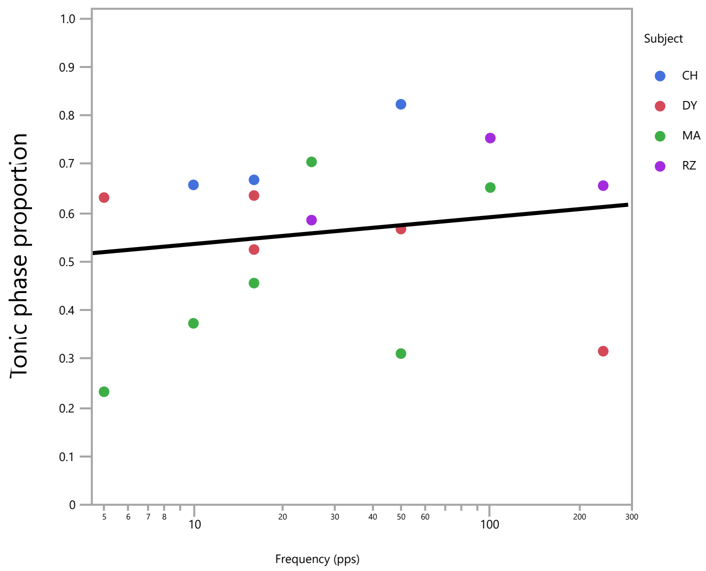

Figure S8. The tonic phase proportion relative to total EMG seizure duration did not vary significantly across stimulus frequency (R^2 = 0.0288, p = 0.515). Colors denote subject and regression line is in black.

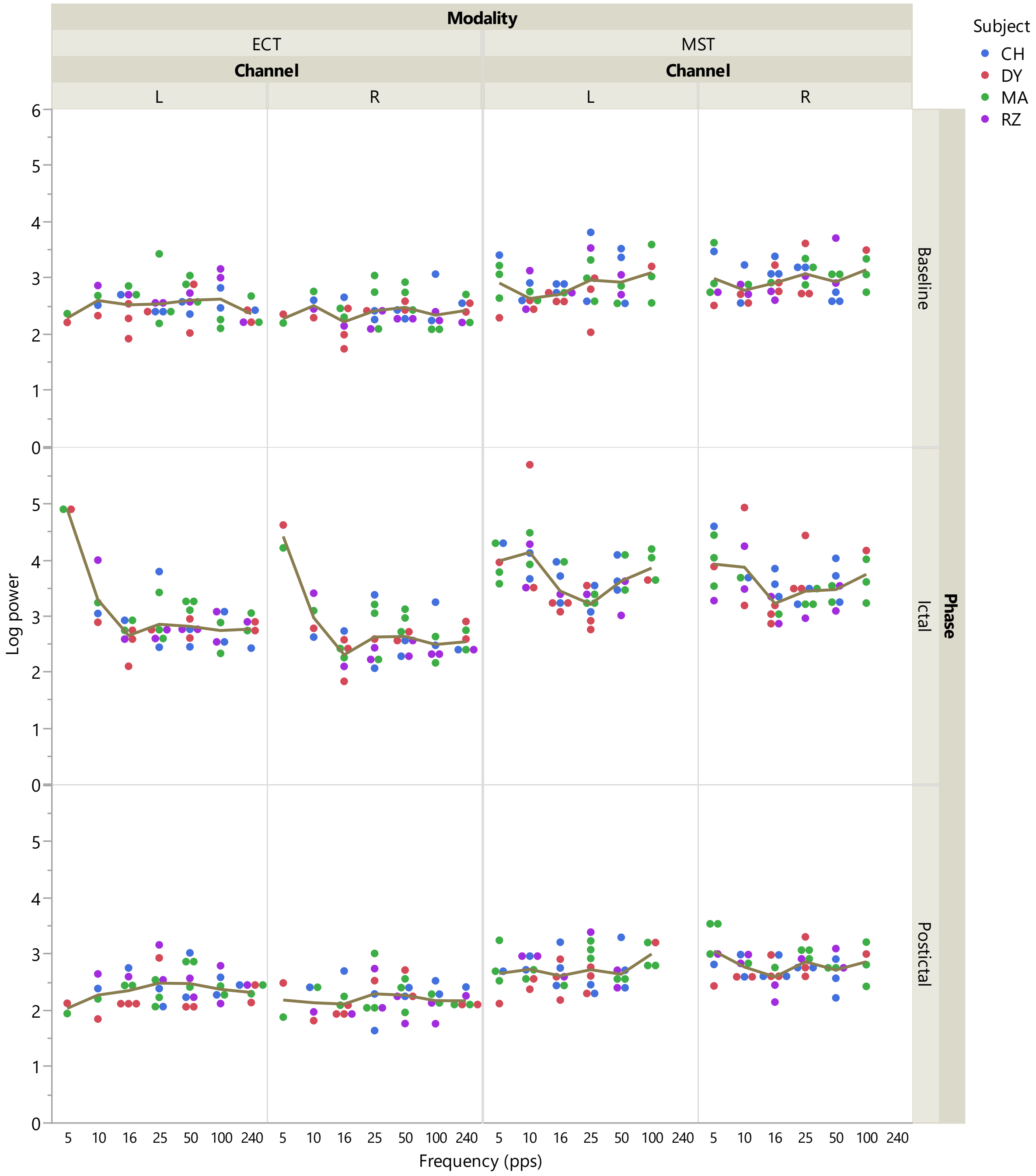

Figure S9. Log-transformed global EEG power across stimulation conditions (modality and frequency), EEG channels (left, L, and right, R), and seizure phase (baseline, ictal, and postictal). Colored dots correspond to data from individual sessions and subjects. Lines show the average within stimulation frequency.

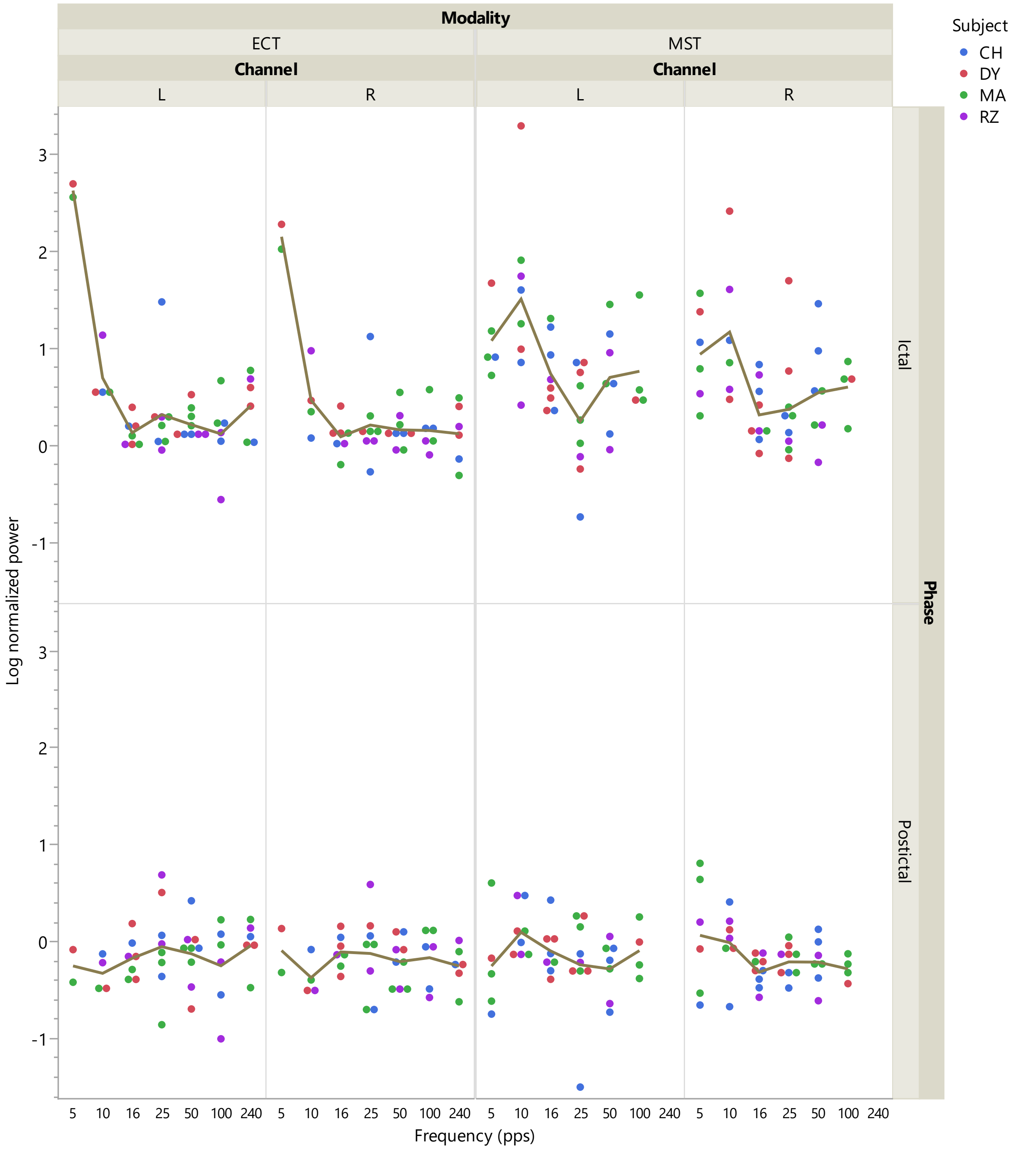

Figure S10. Log-transformed global EEG power normalized to baseline across stimulation conditions, EEG channels, and seizure phase. Display conventions as in Figure S9.

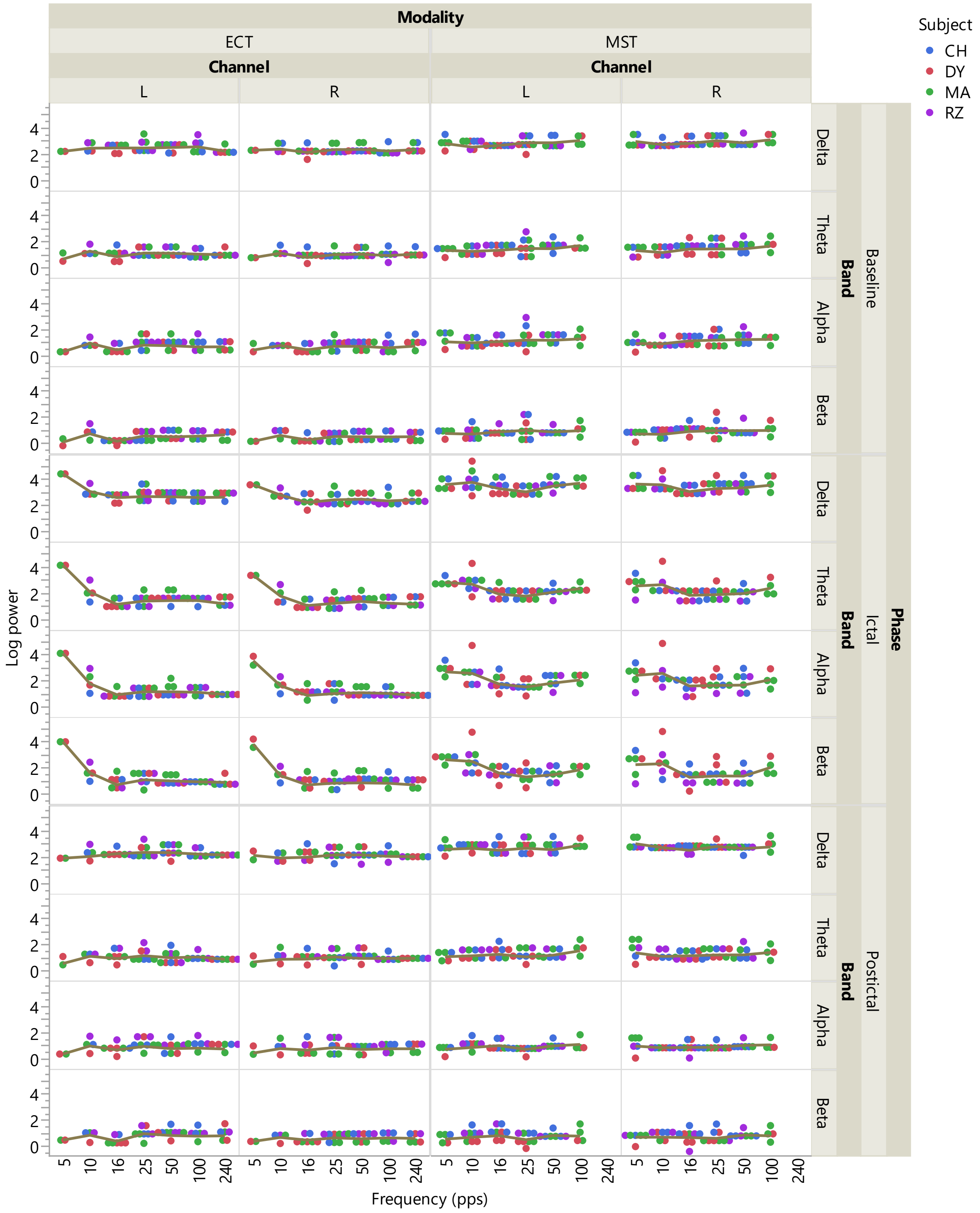

Figure S11. Log-transformed EEG power across stimulation conditions, EEG channels, seizure phase, and EEG bands. Display conventions as in Figure S9.

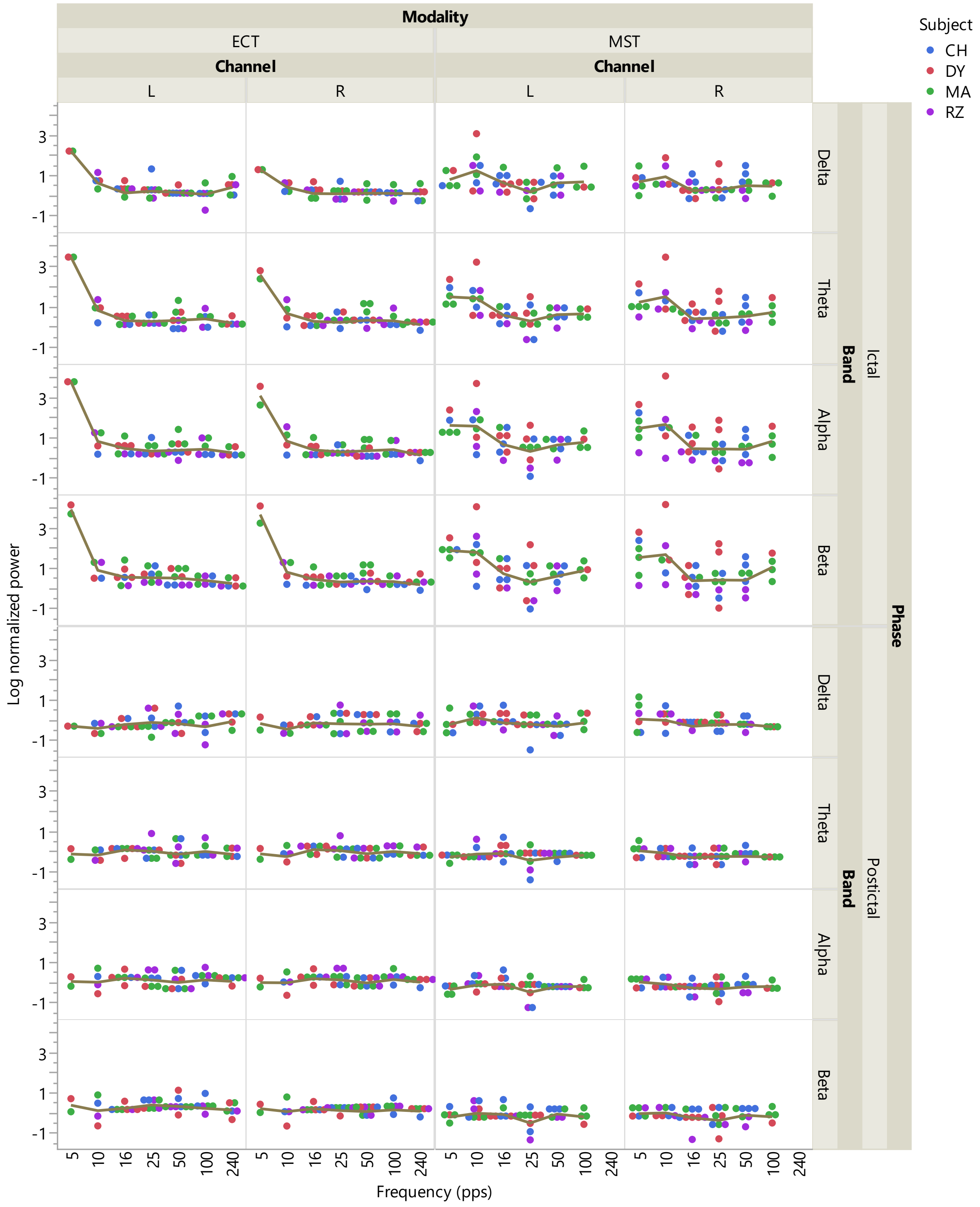

Figure S12. Log-transformed EEG power normalized to baseline across stimulation conditions, EEG channels, seizure phase, and EEG bands. Display conventions as in Figure S9.

Additional discussion

Stimulus train duration

We observed reduction of the train duration at ST with increasing frequency, even though more pulses were delivered at higher frequencies (Figure 2B). Similar observations in other nonhuman animal studies led some researchers to conclude that stimulus frequencies above 100 pps induce seizures more efficiently than lower frequencies (2-5). These results contradict human ECT and MST studies that suggest lower frequencies are more efficient (6-11). This discrepancy may stem from the fact that in some of the animal studies frequency and number of pulses were varied simultaneously since frequency was evaluated for a fixed train duration (2, 3, 12). Thus, an increase in frequency was always associated with a proportional increase of the number of pulses. The probability of a seizure increases as more pulses are delivered (13-15); consequently, higher frequencies will be biased to lower ST. Nevertheless, these prior studies contain clues that lower frequencies may be more efficient if the number of pulses were held constant, as the simultaneous increase of both frequency and number of pulses produced slowing incremental reduction of ST, and eventually the threshold increased at high frequencies (>> 300 pps).

EEG comparison to prior study

Differences in seizure expression between ECT and MST in nonhuman primates have been discussed previously. Cycowicz et al. (2018) reported a randomized experiment where 24 macaca mulatta (12 male) received 6 weeks of daily 50 Hz (100 pps) ECS or 50 Hz (50 pps) MST (16). Threshold seizure induction resulted in differential EEG power distribution during the ictal and postictal periods. Specifically, ictal EEG power for ECT increased progressively from the low (delta) to high (beta) frequency bands; MST induced seizures had higher delta power, but lower beta power compared to ECS seizures. In the postictal period, ECT produced robust suppression of EEG power in the delta-to-alpha frequency bands, but less suppression of beta power, while MST produced slight suppression of theta-to-beta power, but no suppression of delta power (16). Figure S13 compares our data with those of Cycowicz et al. In the present work, ECT was delivered at individualized amplitude of 364 ± 148 mA, less than half the amplitude (800 mA) used by Cycowicz et al. Further differences between the present study and Cycowicz et al. included the electrode placement (unilateral versus bilateral), shorter pulse width (0.2 ms versus 0.5 ms),

and older nonhuman primate subjects (13.32 ± 2.73 years versus 2.83 ± 0.46 years). Compared to Cycowicz et al., MST was also delivered at a different amplitude in the present study (78 ± 9% of maximum device output, MSO, compared to 100% MSO) and with a different device (MagPro versus Magstim). Presumably due to these differences, the ECT ictal power and postictal suppression are greatly reduced in the present work, whereas the MST ictal seizure expression is reduced to a lesser degree and the postictal suppression is increased. Nonetheless, the different shape of the ictal power distribution across the EEG bands between ECT and MST is similar between the two studies, suggesting potential mechanistic differences between the two modalities.

Finally, both datasets in Figure S13 are at seizure threshold (ST), whereas clinical treatments are conventionally administered above ST. The present study did not explore suprathreshold stimulation, while Cycowicz et al. did, finding that increasing the train duration, and hence the number of pulses, by 150% relative to the ST, enhanced the ictal EEG power in the theta, alpha, and beta band and post-ictal suppression in the theta and alpha band for ECT but not MST. This points to potentially important further interactions between stimulation modality and the stimulus parameters.

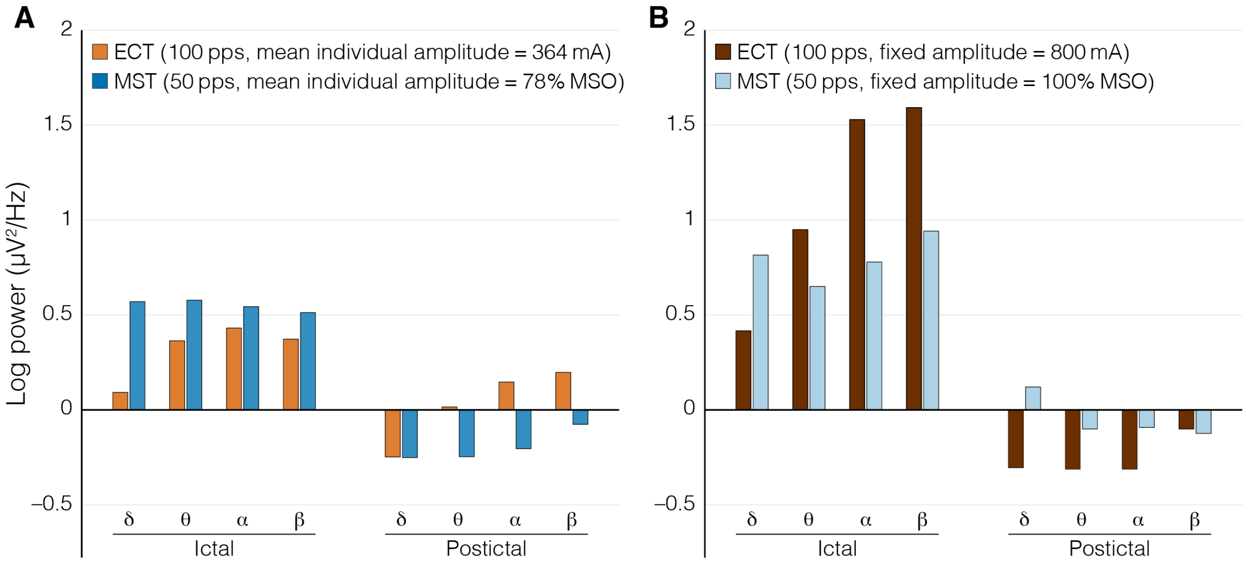

Figure S13. Ictal and postictal EEG power normalized to pre-stimulus baseline power for ECT with 100 pps and MST with 50 pps: A) The present work and B) Cycowicz et al., 2018 (16). In the present work, ECT was delivered at individualized amplitude of 364 ± 148 mA; MST was

delivered at individualized amplitude of 78 ± 9% maximum stimulator output (MSO). Cycowicz et al. delivered ECT at fixed amplitude of 800 mA and MST at fixed amplitude of 100% MSO.

Putative physiological mechanisms

We demonstrated that stimulus trains in the 10−25 pps range are most efficient for inducing seizures, and the ST increases sharply at lower frequencies and gradually at higher frequencies. This finding is largely consistent with evidence from in vitro, animal, clinical, and computational studies that offer insights into potential mechanisms. Electrical stimulation of rat hippocampal slices with 10 pps for 2–3 s preceded by 40 pps for 1 s (17) or 100 pps for 0.2–0.4 s (18, 19) results in prolonged depolarization of pyramidal cells. Longer stimulation with 3–10 pps for 30–60 s (20) or 60 pps for 2 s (21) can trigger epileptiform activity. The proposed mechanism was a reduction in GABA-A-mediated inhibitory postsynaptic potentials associated with an increase in the concentration of extracellular K+ and intracellular Cl− (17-20). Increased extracellular K+ concentration attenuates hyperpolarizing/inhibitory currents in surrounding neurons, and increased intracellular Cl− concentration causes a switch of GABA-A receptor function from inhibitory to excitatory (22, 23). Further, changes in K+ can trigger transitions between different neural activity patterns, including from resting state to single spike firing or to depolarization block (24-26). Very low stimulus frequencies (< 1 Hz) produce only small neuromodulatory effects in the brain (27, 28), and there is an opportunity for inhibitory currents to operate to restore equilibrium (29).

Another important observation is that electrical stimulation at both low (~ 1 pps) or high frequencies (~ 130 pps) can suppress ongoing epileptic activity (29-31). Repetitive transcranial magnetic stimulation (rTMS) pulse trains delivered at 1 pps are inhibitory (32), and rTMS at 1 or 5 pps attenuated several EEG markers of penicillin-induced seizure activity in rats, while trains delivered at 10 pps facilitated the seizure (33). The anti-seizure mechanisms of low frequency stimulation involve long-lasting hyperpolarization mediated by GABA-B inhibitory postsynaptic potentials and slow afterhyperpolarization (29). Putative mechanisms of high frequency stimulation are extensions of the refractory period, normally less than 2 ms in central neurons (34), and induction of intermittent axonal block and neural firing desynchronization (35-38). Thalamic stimulation in epilepsy patients at 15–45 pps produced synchronization of hippocampal

local field potentials, whereas higher frequencies above 45 pps promoted desynchronization of hippocampal activity and reduction of pathological epileptic discharges (30). Adenosine release could be another factor mediating seizure suppression at higher frequencies (23, 29, 39-41).

It is also notable that the optimal frequencies for seizure induction suggested by our results correspond well with EEG frequencies observed during the onset of epileptic seizures. Gastaut and Broughton described a 10 Hz "recruiting rhythm" in the EEG during the first 10 seconds of generalized tonic-clonic seizures, followed by a progressive decrease in frequency until seizure termination (42). There has been little investigation of the mechanisms underlying the recruiting rhythm, even in animal models (43). In vitro studies have shown bursting patterns of ~ 10 Hz in groups of neurons in response to epileptogenic stimuli, which may result from sustained dendritic depolarization (44, 45). Gastaut and Broughton likened the rhythm to that seen in thalamically-driven cortical responses (42); whether thalamocortical interactions play a role in seizure induction by ECT or MST is currently unknown. Perhaps a "resonance" effect may exist whereby fewer stimulus pulses are required to induce seizures at frequencies characteristic of the onset of endogenous epileptic activity.

Finally, our EEG analysis found stimulus frequency affected ictal expression, suggesting shifts in the dynamics of neural inhibitory and excitatory processes with stimulation frequency.

Implications for clinical ECT and MST

The present study evaluated the impact of ECT and MST stimulus frequency on ST and seizure characteristics at threshold in nonhuman primates. The effects of stimulus frequency on the characteristics of suprathreshold seizure induction, therapeutic efficacy, and side effects has to be investigated in future studies. Evaluating efficacy would require appropriately designed clinical trials, since animal models of depression have significant limitations (46). Presently, the literature on the impact of stimulus frequency on clinical outcomes of ECT and MST is limited. Studies that considered the effect of stimulus frequency on the efficiency of seizure induction with ECT found that higher frequencies were less efficient (6-9, 13), consistent with our results. In one ECT study, stimulus frequency had no effect on heart rate or seizure induction with suprathreshold trains with matched number of pulses at 30 Hz (60 pps) and 60 Hz (120 pps) (47). Another trial found no differences in seizure durations, ictal cardiovascular responses, and therapeutic outcome between

the 50 pps and 200 pps (48). A third study indicated that 40 Hz (80 pps) is more effective than 100 Hz (200 pps), and there were no significant differences in memory testing (49).

Stimulus frequency may impact clinical MST outcomes (10, 50). An MST trial by Daskalakis et al. comparing low (25 pps), medium (50 or 60 pps), and high (100 pps) frequency used a titration of the individual ST by incrementing the number of pulses similar to the present study, except that the pulse amplitude was fixed at MSO for all participants (10). Treatments were then delivered by increasing the number of pulses relative to the ST for effective suprathreshold stimulation (10, 11). Notably, there were substantial differences in the average number of pulses delivered during treatment for each group: 396, 724, and 753, for low, medium, and high frequency, respectively (11), reflecting lower STs for the low frequencies, consistent with our findings. Regarding efficacy, high frequency produced the highest rates of remission of depression symptoms, followed by moderate and low frequency (10). However, neither response rates nor number of treatments associated with remission were significantly different across the conditions (10). Since 100 pps MST was administered with nearly twice the number of pulses for 25 pps MST, it is unclear if the superior remission rate was caused by the higher frequency or the larger number of pulses. The fact that response rates at 100 pps and 25 pps were comparable despite the two-fold difference in number of pulses, leaves open the possibility that 25 pps could be as effective or superior if the number of pulses were matched. Further, while seizure duration was comparable across the stimulus frequency groups (10), consistent with our findings, a common EEG measure of seizure adequacy was better for 25 pps and 50 pps than for 100 pps (11). On the other hand, the time to reorientation was significantly shorter for the high frequency than the medium or low frequency stimulation, and consistency of autobiographical recall was more affected for the medium frequency than low or high frequency, suggesting that higher frequency stimulation could have an advantageous cognitive side effects profile (10). Interestingly, an exploratory analysis suggested that the low and medium stimulus frequencies may be more effective that the high frequency at reducing suicidality (51), potentially pointing to interactions between stimulus frequency and symptom dimensions.

These findings from clinical studies, while limited, hint at interesting possibilities about the impact of stimulation frequency on seizure characteristics, efficacy, and side effects, but more research is needed. Our results on the relationship between the ECT and MST stimulus frequency and ST can inform such work. One practical challenge is testing low frequency stimuli

with an adequate number of pulses. In our data, frequencies above 10 pps required stimulation duration below 10 s, which is practical. However, prior findings that for ECT to be effective the number of stimulus pulses has to exceed the ST by several times (52) would imply relatively long stimulus trains that may surpass what commercially available devices can deliver: maximum of 8 s in modern ECT devices (53-55) and 20 s in MST devices (10). Therefore, devices capable of extended trains may be necessary to investigate effective low-frequency stimulation, and the safety of longer train duration has to be assessed. Our preliminary results in nonhuman primates indicate that long trains of 25 pps with individually-titrated current amplitude and lasting up to 40 s appear safe based on heart rate, blood pressure, and blood oxygenation level assessment (15).

Implications for rTMS

Our data on ST across stimulus frequencies could potentially inform safety considerations for rTMS, where seizures must be avoided. While there is the potential confound of anesthesia, the anesthetic agents used in the present study are common in ECT and are selected to not increase the ST significantly. With regard to rTMS safety, the assumption has been that higher frequencies carry higher seizure risk (56, 57). The decreasing ST as frequency increases in the 5–25 pps range is consistent with the guidelines for rTMS which specify that the number of pulses that can be safely administered without inducing a seizure decreases similarly in the 1–25 pps frequency range (56). However, our results suggest that above 25 pps, the rTMS ST may start to increase; this may contribute to the documented safety of theta-burst stimulation which incorporates 50 pps bursts (57) and could inform safety considerations for protocols involving very high frequencies (58, 59).

References

1. Conradsen I, Moldovan M, Jennum P, Wolf P, Farina D, Beniczky S (2013): Dynamics of muscle activation during tonic-clonic seizures. *Epilepsy Res*. 104:84-93.
2. Liberson WT (1945): Time factors in electric convulsive therapy. *Yale J Biol Med*. 17:571-578.
3. Hovorka EJ, Schumsky DA, Work MS (1960): Electroconvulsive thresholds as related to stimulus parameters of unidirectional ECS. *J Comp Physiol Psychol*. 53:412-414.
4. Weaver L, Ravaris C, Rush S, Paananen R (1974): Stimulus parameters in electroconvulsive shock. *J Psychiatr Res*. 10:271-281.
5. Hyrman V, Palmer LH, Cernik J, Jetelina J (1985): ECT: the search for the perfect stimulus. *Biol Psychiatry*. 20:634-645.

6. Swartz CM, Larson G (1989): ECT stimulus duration and its efficacy. *Ann Clin Psychiatry*. 1:147-152.
7. Devanand DP, Lisanby SH, Nobler MS, Sackeim HA (1998): The relative efficiency of altering pulse frequency or train duration when determining seizure threshold. *J ECT*. 14:227-235.
8. Girish K, Gangadhar BN, Janakiramaiah N, Lalla RK (2003): Seizure threshold in ECT: effect of stimulus pulse frequency. *J ECT*. 19:133-135.
9. Weaver LA, Jr., Ives J, Williams R (1982): Studies in brief-pulse electroconvulsive therapy: the voltage threshold, interpulse interval, and pulse polarity parameters. *Biol Psychiatry*. 17:1131-1143.
10. Daskalakis ZJ, Dimitrova J, McClintock SM, Sun Y, Voineskos D, Rajji TK, et al. (2020): Magnetic seizure therapy (MST) for major depressive disorder. *Neuropsychopharmacology*. 45:276-282.
11. Backhouse FA, Noda Y, Knyahnytska Y, Farzan F, Downar J, Rajji TK, et al. (2018): Characteristics of ictal EEG in Magnetic Seizure Therapy at various stimulation frequencies. *Clin Neurophysiol*. 129:1770-1779.
12. Woodbury LA, Swinyard CA (1952): Stimulus parameters for electroshock seizures in rats. *Am J Physiol*. 170:661-667.
13. Peterchev AV, Rosa MA, Deng ZD, Prudic J, Lisanby SH (2010): Electroconvulsive therapy stimulus parameters: rethinking dosage. *J ECT*. 26:159-174.
14. Alexander L (1953): *Treatment of mental disorder*. Philadelphia: W. B. Saunders and Co.
15. Peterchev AV, Sikes-Keilp C, Koval M, Lisanby SH (2016): Re-evaluating the Electroconvulsive Therapy Stimulus: Train Duration. *Biological Psychiatry*. 79:401S.
16. Cycowicz YM, Rowny SB, Luber B, Lisanby SH (2018): Differences in Seizure Expression Between Magnetic Seizure Therapy and Electroconvulsive Shock. *J ECT*. 34:95-103.
17. Bracci E, Vreugdenhil M, Hack SP, Jefferys JGR (2001): Dynamic modulation of excitation and inhibition during stimulation at gamma and beta frequencies in the CA1 hippocampal region. *J Neurophysiol*. 85:2412-2422.
18. Bracci E, Vreugdenhil M, Hack SP, Jefferys JGR (1999): On the synchronizing mechanisms of tetanically induced hippocampal oscillations. *J Neurosci*. 19:8104-8113.
19. Kaila K, Lamsa K, Smirnov S, Taira T, Voipio J (1997): Long-lasting GABA-mediated depolarization evoked by high-frequency stimulation in pyramidal neurons of rat hippocampal slice is attributable to a network-driven, bicarbonate-dependent K+ transient. *J Neurosci*. 17:7662-7672.
20. Thompson SM, Gahwiler BH (1989): Activity-dependent disinhibition. 1. Repetitive stimulation reduces IPSP driving force and conductance in the hippocampus in vitro. *J Neurophysiol*. 61:501-511.
21. Stasheff SF, Bragdon AC, Wilson WA (1985): Induction of epileptiform activity in hippocampal slices by trains of electrical stimuli. *Brain Res*. 344:296-302.
22. American Epilepsy Society (2006): *An introduction to epilepsy*. Bethesda, MD: American Epilepsy Society.
23. Boison D (2013): Adenosine and seizure termination: endogenous mechanisms. *Epilepsy Curr*. 13:35-37.
24. Barreto E, Cressman JR (2011): Ion concentration dynamics as a mechanism for neuronal bursting. *J Biol Phys*. 37:361-373.

25. Wu XX, Shuai JW (2012): Multistability in a neuron model with extracellular potassium dynamics. *Phys Rev E Stat Nonlin Soft Matter Phys*. 85:061911.
26. Contreras SA, Schleimer JH, Gulledge AT, Schreiber S (2021): Activity-mediated accumulation of potassium induces a switch in firing pattern and neuronal excitability type. *PLoS Comput Biol*. 17:e1008510.
27. Julkunen P, Saisanen L, Hukkanen T, Danner N, Kononen M (2012): Does second-scale intertrial interval affect motor evoked potentials induced by single-pulse transcranial magnetic stimulation? *Brain Stimul*. 5:526-532.
28. Pellicciari MC, Miniussi C, Ferrari C, Koch G, Bortoletto M (2016): Ongoing cumulative effects of single TMS pulses on corticospinal excitability: An intra- and inter-block investigation. *Clin Neurophysiol*. 127:621-628.
29. Toprani S, Durand DM (2013): Long-lasting hyperpolarization underlies seizure reduction by low frequency deep brain electrical stimulation. *J Physiol*. 591:5765-5790.
30. Yu T, Wang X, Li Y, Zhang G, Worrell G, Chauvel P, et al. (2018): High-frequency stimulation of anterior nucleus of thalamus desynchronizes epileptic network in humans. *Brain*. 141:2631-2643.
31. Durand DM, Bikson M (2001): Suppression and control of epileptiform activity by electrical stimulation: A review. *Proc IEEE*. 89:1065-1082.
32. Di Lazzaro V, Dileone M, Pilato F, Capone F, Musumeci G, Ranieri F, et al. (2011): Modulation of motor cortex neuronal networks by rTMS: comparison of local and remote effects of six different protocols of stimulation. *J Neurophysiol*. 105:2150-2156.
33. Lin CY, Li K, Franic L, Gonzalez-Martinez J, Lin VW, Najm I, et al. (2014): Frequency-dependent effects of contralateral repetitive transcranial magnetic stimulation on penicillin-induced seizures. *Brain Res*. 1581:103-116.
34. Jankowska E, Kaczmarek D, Hammar I (2022): Long-term modulation of the axonal refractory period. *Eur J Neurosci*. 56:4983-4999.
35. Wilson D, Moehlis J (2015): Clustered Desynchronization from High-Frequency Deep Brain Stimulation. *PLoS Comput Biol*. 11:e1004673.
36. Feng Z, Yu Y, Guo Z, Cao J, Durand DM (2014): High frequency stimulation extends the refractory period and generates axonal block in the rat hippocampus. *Brain Stimul*. 7:680-689.
37. Yuan Y, Feng Z, Yang G, Ye X, Wang Z (2022): Suppression of Neuronal Firing Following Antidromic High-Frequency Stimulations on the Neuronal Axons in Rat Hippocampal CA1 Region. *Front Neurosci*. 16:881426.
38. Guo Z, Feng Z, Wang Y, Wei X (2018): Simulation Study of Intermittent Axonal Block and Desynchronization Effect Induced by High-Frequency Stimulation of Electrical Pulses. *Front Neurosci*. 12:858.
39. Crosson CE, Gray T (1997): Response to prejunctional adenosine receptors is dependent on stimulus frequency. *Curr Eye Res*. 16:359-364.
40. Kakiuchi S, Rall TW, McIlwain H (1969): The effect of electrical stimulation upon the accumulation of adenosine 3',5'-phosphate in isolated cerebral tissue. *J Neurochem*. 16:485-491.
41. Loscher W, Kohling R (2010): Functional, metabolic, and synaptic changes after seizures as potential targets for antiepileptic therapy. *Epilepsy Behav*. 19:105-113.
42. Gastaut H, Broughton R (1972): *Epileptic seizures: clinical and electrographic features, diagnosis and treatment*. Springfield, Ill.: Thomas.

43. Kohsaka S, Mizukami S, Kohsaka M, Shiraishi H, Kobayashi K (2002): Widespread activation of the brainstem preceding the recruiting rhythm in human epilepsies. *Neuroscience*. 115:697-706.
44. Kawaguchi Y (2001): Distinct firing patterns of neuronal subtypes in cortical synchronized activities. *J Neurosci*. 21:7261-7272.
45. Traub RD, Borck C, Colling SB, Jefferys JG (1996): On the structure of ictal events in vitro. *Epilepsia*. 37:879-891.
46. Kritzer MD, Peterchev AV, Camprodon JA (2023): Electroconvulsive Therapy: Mechanisms of Action, Clinical Considerations, and Future Directions. *Harv Rev Psychiatry*. 31:101-113.
47. Swartz CM, Manly DT (2000): Efficiency of the stimulus characteristics of ECT. *Am J Psychiatry*. 157:1504-1506.
48. Kotresh S, Girish K, Janakiramaiah N, Rao GU, Gangadhar BN (2004): Effect of ECT stimulus parameters on seizure physiology and outcome. *J ECT*. 20:10-12.
49. Roepke S, Luborzewski A, Schindler F, Quante A, Anghelescu I, Heuser I, et al. (2011): Stimulus pulse-frequency-dependent efficacy and cognitive adverse effects of ultrabrief-pulse electroconvulsive therapy in patients with major depression. *J ECT*. 27:109-113.
50. Kayser S, Wagner S (2018): Stimulation frequency of magnetic seizure therapy contributes to the adequacy of seizures. *Clin Neurophysiol*. 129:1718-1719.
51. Weissman CR, Blumberger DM, Dimitrova J, Throop A, Voineskos D, Downar J, et al. (2020): Magnetic Seizure Therapy for Suicidality in Treatment-Resistant Depression. *JAMA Netw Open*. 3:e207434.
52. Sackeim HA, Prudic J, Devanand DP, Nobler MS, Lisanby SH, Peyser S, et al. (2000): A prospective, randomized, double-blind comparison of bilateral and right unilateral electroconvulsive therapy at different stimulus intensities. *Arch Gen Psychiatry*. 57:425-434.
53. MECTA Corp. (1997): *spECTrum: MECTA Instruction Manual: 100 Joules Domestic*. Lake Oswego, OR, USA: MECTA Corporation.
54. Somatics LLC (2021): *User Manual: Thymatron(R) System IV, Rev. 22*. Venice, FL, USA: Somatics LLC.
55. SigmaStim (2022): *Σigma Instruction Manual Book One: Use of the Device (DOM): 100 Joule*. Tualatin, OR, USA: SigmaStim Domestic LLC.
56. Rossi S, Hallett M, Rossini PM, Pascual-Leone A (2009): Safety, ethical considerations, and application guidelines for the use of transcranial magnetic stimulation in clinical practice and research. *Clin Neurophysiol*. 120:2008-2039.
57. Rossi S, Antal A, Bestmann S, Bikson M, Brewer C, Brockmoller J, et al. (2021): Safety and recommendations for TMS use in healthy subjects and patient populations, with updates on training, ethical and regulatory issues: Expert Guidelines. *Clin Neurophysiol*. 132:269-306.
58. Jung NH, Gleich B, Gattinger N, Hoess C, Haug C, Siebner HR, et al. (2016): Quadri-Pulse Theta Burst Stimulation using Ultra-High Frequency Bursts - A New Protocol to Induce Changes in Cortico-Spinal Excitability in Human Motor Cortex. *PLoS One*. 11:e0168410.
59. Jung NH, Gleich B, Gattinger N, Kalb A, Fritsch J, Asenbauer E, et al. (2021): Double-Sine-Wave Quadri-Pulse Theta Burst Stimulation of Precentral Motor Hand Representation Induces Bidirectional Changes in Corticomotor Excitability. *Front Neurol*. 12:673560.
